# A Cloud-Based IoT Platform for Precision Control of Soilless Greenhouse Cultivation

**DOI:** 10.3390/s21010223

**Published:** 2020-12-31

**Authors:** Alaa Sagheer, Maged Mohammed, Khaled Riad, Mohammed Alhajhoj

**Affiliations:** 1 College of Computer Sciences and Information Technology, King Faisal University, Al-Ahsa 31982, Saudi Arabia; kriad@kfu.edu.sa; 2 Center for Artificial Intelligence and Robotics (CAIRO), Aswan University, Aswan 81582, Egypt; 3 Date Palm Research Center of Excellence, King Faisal University, Al-Ahsa 31982, Saudi Arabia; memohammed@kfu.edu.sa; 4 Department of Agricultural Engineering, Faculty of Agriculture, Menoufia University, Shebin ElKoum 32514, Egypt; 5 Department of Mathematics, Faculty of Science, Zagazig University, Zagazig 44519, Egypt; 6 Department of Arid Land Agriculture, College of Agricultural and Food Sciences, King Faisal University, Al-Ahsa 31982, Saudi Arabia; malhajhoj@kfu.edu.sa

**Keywords:** Internet of Things, smart farming, water productivity, microclimate control, cucumber, arid region, wireless control

## Abstract

Food security has become an increasingly important challenge for all countries globally, particularly as the world population continues to grow and arable lands are diminishing due to urbanization. Water scarcity and lack of labor add extra negative influence on traditional agriculture and food production. The problem is getting worse in countries with arid lands and harsh climate, which exacerbates the food gap in these countries. Therefore, smart and practical solutions to promote cultivation and combat food production challenges are highly needed. As a controllable environment, greenhouses are the perfect environment to improve crops’ production and quality in harsh climate regions. Monitoring and controlling greenhouse microclimate is a real problem where growers have to deal with various parameters to ensure the optimal growth of crops. This paper shows our research in which we established a multi-tier cloud-based Internet of Things (IoT) platform to enhance the greenhouse microclimate. As a case study, we applied the IoT platform on cucumber cultivation in a soilless medium inside a commercial-sized greenhouse. The established platform connected all sensors, controllers, and actuators placed in the greenhouse to provide long-distance communication to monitor, control, and manage the greenhouse. The implemented platform increased the cucumber yield and enhanced its quality. Moreover, the platform improved water use efficiency and decreased consumption of electrical energy. Based on the positive impact on water use efficiency and enhancement on cucumber fruit yield and quality, the established platform seems quite suitable for the soilless greenhouse cultivation in arid regions.

## 1. Introduction

As the world population continues to grow, the rising demand for food and crop production is significant. The United Nations estimated the current world population as 7.7 billion and is expected to approach 8.6 billion in 2030 and 9.8 billion in 2050 [1]. It is expected that by 2050 we will need 42% more cropland and 120% more water to feed the world population [2,3]. If we add other challenges, such as water scarcity and lack of labor, we will realize to what extent the difficulties that traditional outdoor agriculture has to face [4]. One optimum solution for boosting the food and crop production is the protected cultivation via what is called greenhouses [5].

The greenhouse is a structure covered with plastic films mainly developed to cultivate the plants inside. It can adjust the plants growing conditions according to the plants need and, therefore, contribute to enhancing the quality and quantity of the crops. Most of the traditional greenhouses, particularly those in countries with arid lands, neglect to control many environmental parameters such as humidity, temperature, among others [6]. Greenhouses usually require a specific number of devices for environmental control; however, they need a standard, accurate monitoring, and management of several environmental parameters [7].

In earlier greenhouse farming, the environmental parameters and the growing conditions were based on the growers’ observations that were accomplished manually, which requires enough numbers of growers [8]. In recent years, the concept of smart farming has been attracted broad attention, which leads to widespread of the greenhouse farming [9,10]. Smart greenhouse farming is an emerging indoor farming that refers to managing the greenhouse using information and communication technologies (ICT) to increase the crops’ quantity and quality while optimizing the human labor required [11,12]. Indeed, developing smart greenhouses ensures rising amounts of crops and fruits.

Toward a high benefit greenhouse farming, the research community moved further toward the adoption of soilless cultivation inside the greenhouse [13,14]. Soilless cultivation is the cultivation of plants in containers that do not include soil. It is widely demonstrated that many growth parameters are affected by the type of soilless culture media [15,16]. From a practical point of view, there are two main types of soilless culture; namely, substrate-culture soilless (SCS) and liquid-culture soilless (LCS) [17]. In the former type, the SCS culture uses either organic or inorganic substrates allowing the plants to have ideal nutrient uptake and adequate growth to optimize the oxygen and water holding [18]. In the other type, the LCS cultivation system could include either aeroponics or hydroponics as a growing medium. These growing media use a mixture of water and nutrients instead of the substrate-culture [19].

It is widely demonstrated that the SCS systems are much better than both the LCS systems and the traditional soil cultivation systems due to that the SCS culture allows for better control of the crop nutrients and water supplies [17]. Ideally, the SCS system is independent of the kind of soil or soil properties. SCS system utilizes an inexpensive irrigation system even with lower quality on the contrary of traditional soil-based cultivation. The impact of an SCS system increases when it uses a closed-drainage system, in which nutrient solution is gathered and recirculated to be utilized in the following irrigation cycles [20]. Meanwhile, the SCS systems are relatively better than the LCS systems because it provides a solid growing medium for the plant compared to the liquid growing medium. Specifically, the SCS medium creates a shield around the plant root to avoid any potential water stress. Moreover, in some cultivation environments, the SCS substrate prevents any stress fluctuations in the root zone to avoid plant damage [21].

All the above-undertaken outdoor agriculture challenges are embodied clearly in Saudi Arabia, a desert country located in a very hot and arid region. Besides, the environmental and weather conditions are not ideal for traditional outdoor agriculture due to severe and harsh climate, extreme temperature degrees, and limited water resources. These reasons cause a food gap in this country, which require practical solutions suitable to the local environmental conditions to enhance the food production [22]. One of the practical solutions is the soilless greenhouse cultivation, in which the crops grow under some preset environmental conditions. This solution significantly mitigates the influence of harsh climate and farmlands scarcity in similar arid regions, which will increase crop and food production [8,23].

### 1.1. Related Work

The success of soilless greenhouse cultivation is based on the collected data accuracy and parameter measurement efficiency [15,24]. Collecting and reporting various measurements on a timely basis are considered the backbone of soilless greenhouse cultivation, particularly with the heterogeneity of farming parameters [25]. The cheapest way to collect data entails the use of different types of specific-purpose wireless actuators and sensors. This is made possible by establishing an IoT infrastructure inside the greenhouse. This allows the interconnection of actuators and sensors via the Internet to communicate effectively and transfer meaningful data [26]. In this way, the growers can monitor the crops steadily and accurately, manage and control the environmental parameters, automate logistic actions taken, and early detect any undesired situations [27,28]. Toward these targets, sincere efforts have been recently exerted to develop different sensing systems for monitoring and controlling greenhouses [29].

Postolache et al. [6] presented a solution for monitoring the water and air quality used in the greenhouse. The authors employed two versions of a distributed-sensing network whose number of nodes depends on the greenhouse area. One of the defects of this system is the necessity to connect a one-by-one sensor node according to the Bluetooth requirements. The implementation did not perform sensors calibration to ensure adequate accuracy of the measured quantities, particularly because the sensors and electronics are operated in field conditions. Patil et al. [30] presented a lightweight Blockchain-based architecture for a smart greenhouse to provide security and privacy of the data exchange. However, the authors presented a theoretical framework and did not apply their proposed architecture to a real greenhouse situation or a specific plant.

Singh et al. [31] developed a microclimatic model of a naturally ventilated greenhouse for the cucumber cropping in a soilless media. They considered heat and mass transportation, such as transpiration, radiation, convection, and natural ventilation, and solved it using their own source-code. But, some coefficients require adjustments in advance concerning the change in the greenhouses’ conditions. Besides, Singh’s model ignored the management and automation of the present greenhouse. Wang et al. [32] designed a data encapsulation method that could be feasibly applied to achieve data communication and data interoperability in a distributed greenhouse IoT system. Wang’s research tested the data communication mechanism for real-time and cumulative data synchronization between the gateway and the server. Subahi et al. [33] introduced an IoT based intelligent system for controlling the temperature inside a greenhouse to reduce energy consumption while maintaining good conditions. The systems use the Petri Net technique by generating the suitable reference temperature, which is sent later on to a temperature regulation phase.

For local related work, Al-Harbi et al. [34] investigated the feasibility of using grafting to improve the yield and quality of the cucumber fruit under water stress conditions in Saudi Arabia. They concluded the positive effects of grafting on plant growth, productivity, and water use efficiency, which support this strategy as a useful tool for improving water stress tolerance in greenhouses. Almohithef et al. [35] presented a survey of plant-parasitic nematode genera associated with certain greenhouse vegetable crops in Saudi Arabia. It is known that the plant-parasitic nematodes are serious pathogens attacking vegetable crops that are grown in domestic greenhouses.

### 1.2. Problem Statement and Motivation

According to recent statistics of the World Bank, Saudi Arabia has only 1.8% of its overall space as arable lands, like many countries in the middle east and Arab region [22]. The total area and production of fruit-bearing vegetables under greenhouse farming in Saudi Arabia reach 3019 ha, and 252,824 ton in 2016, respectively [35]. Cucumber is the second, where Tomato is the first, main greenhouse fruits-bearing vegetables grown in Saudi Arabia, with a cultivated area of 885 ha and a total production of 79,650 tons, respectively [35,36]. Yet, number of local studies that have concerned with soilless greenhouse cultivation still represent a shortcoming for our local research community. By giving due consideration to traditional agriculture challenges and increasing demands, intensive research efforts should be exerted to investigate possible alternative ways to enhance soilless greenhouses cultivation in this region of the world.

This motivated us to accomplish the current research in which we established an integrated greenhouse cultivation system using substrate-culture soilless. The established cultivation system comprises a multi-tier cloud-based IoT platform that connects all sensors, controllers, and actuators placed in the greenhouse. The platform established a long-distance communication to monitor, control, and manage the greenhouse microclimate and all its logistic systems, including the irrigation system, and the drainage systems. To evaluate the established cultivation system, we applied it on the cucumber fruit planted in a substrate-culture soilless medium. The established platform is completely instantiated in a commercial-sized greenhouse, with area as 56 m × 45 m located at the Research and Training Station of King Faisal University. The implemented platform increased the cucumber yield and quality, improved water use efficiency, and decreased electrical energy consumption.

We can briefly summarize the main contributions of the current research as follows:

- Establish a multi-tier cloud-based IoT platform for monitoring, controlling, and managing the greenhouse microclimate.
- Implement the established platform in precision control of soilless greenhouse cultivation (cucumber as a case study) in arid regions.
- Investigate impact of the overall established cultivation system on the crop yield and product quality.
- Improve water use efficiency and electrical energy productivity.

## 2. Material and Methods

### 2.1. Greenhouse Description

**Figure 1:**
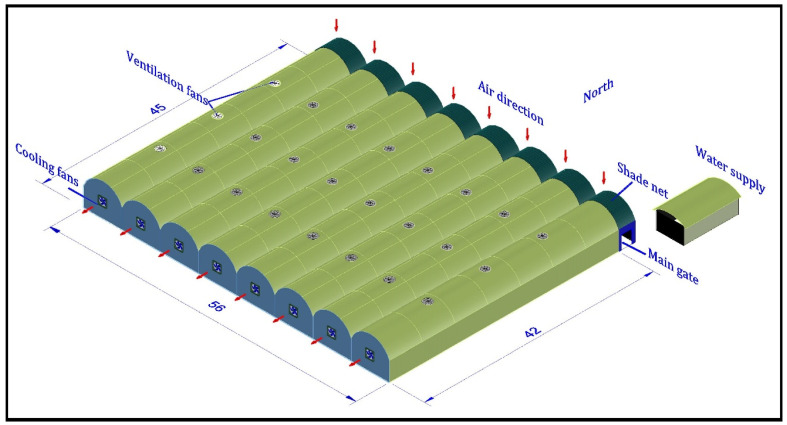
A diagram for the greenhouse from outside with all logistic facilities.

The current research was conducted between the middle of August and the middle of November 2020, in a commercial-sized greenhouse at the Agricultural Training and Research Station, King Faisal University, KSA (Latitude: 25.270142° N, Longitude: 49.708259° E). Figure 1 shows a diagram of the greenhouse from outside including all logistic facilities. The figure demonstrates that the greenhouse has an arch roof (multi-span), which facilitates the possibility of regulating the greenhouse microclimate plus its high resistance to strong winds. The actual area of the greenhouse is 2520 m${}^{2}$ with dimensions of 56 m × 45 m. The greenhouse structure consists of columns, beams, and brackets made of galvanized iron tubes with 25 cm thickness and a diameter of 5 cm, assembled together by screws and nuts. The maximum height of the greenhouse is 6 m, whereas the sidewalls have a height of 3.5 m.

As depicted in Figure 1, the greenhouse contains eight tunnels, each with a width of 7 m, and contains four cultivation lines. The greenhouse walls are covered by 0.8 mm thickness corrugated polycarbonate with a light transmission of 90% and diffusion of 40% while the top surface is roofed by polycarbonate and clear 6-mil polyethylene film. In addition, the greenhouse roof is equipped with a lighting system and an electrically regulated shading network to regulate the intensity of lighting inside the greenhouse and to reduce the overall temperature when needed. The ventilation inside the greenhouse is established by 24 roof-mounted exhaust fans, each of 50 cm diameter and a power of 300 Watt. The greenhouse evaporative cooling is regulated by eight wall-mounted fans, each of 150 cm diameter and a power of 750 Watt, and a cellulose evaporating cooling pad with a thickness of 10 cm and a total area of 75 m${}^{2}$. For regulating the relative humidity in the greenhouse, eight fogging systems were used each with a power of 150 Watt.

**Figure 2:**
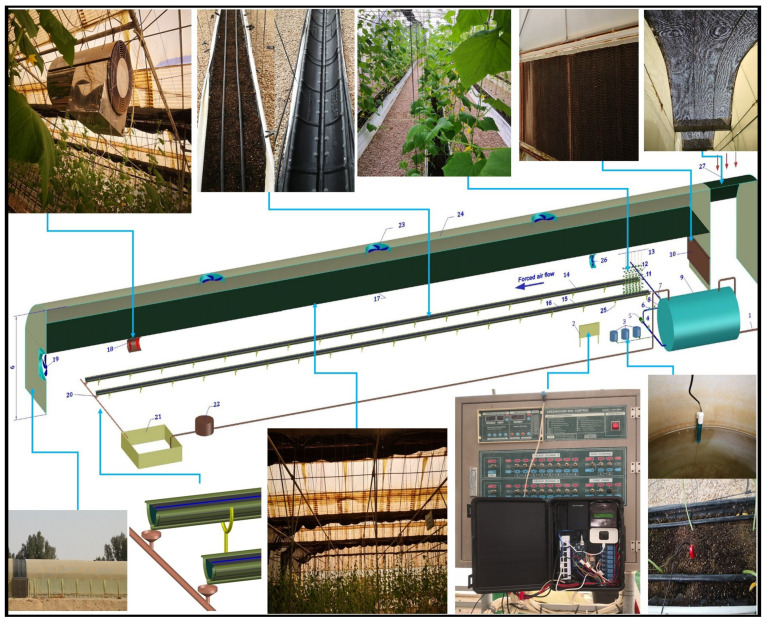
An interior view for a cultivation line in the greenhouse with different irrigation, drainage, and control systems, where: 1: Water supply, 2: Control panel, 3: Fertilizer tanks, 4: Filter, 5: Irrigation pump, 6: Pressure regulator, 7: Electrical valve, 8: Flow meter, 9: Water tank, 10: Pad cooling, 11: Plants, 12: Wire, 13: Trellising cable, 14: Drip irrigation tubing, 15: Oval shape trough, 16: Peat moss media, 17: Shading scree, 18: Air heater, 19: Cooling fan, 20: Collection funnel, 21: Drainage water tank, 22: Filter, 23: Ventilation fan, 24: Clear 6-mil polyethylene film, 25: UV-treated troughs, 26: Fogger fan, and 27: Shading screen.

In each cultivation line, we used many troughs (treated against ultraviolet light) with an oval shape for reducing the quantity of the growth medium. The size of each trough is 18 cm in depth, 30 cm in width, and 90 cm in length. The troughs are interlocked together to create a line with 35 m long. The cultivation line contains around 40 troughs with a total of about 200 plants. The space between any two cultivation lines is 175 cm. Each cultivation line is supplied with a separate irrigation and drainage unit. It should be mentioned that, the troughs have a bottom longitudinal drainage channel to drain the extra water in case of any technical problem. The drainage water is collected at the end of each line through a collection funnel, which is connected to the drainage network of the whole greenhouse. Finally, the drainage water is collected in a specific tank to be filtered, sterilized, and reused in the following closed irrigation cycle. Figure 2 shows the elements of each cultivation line.

### 2.2. System Architecture

**Figure 3:**
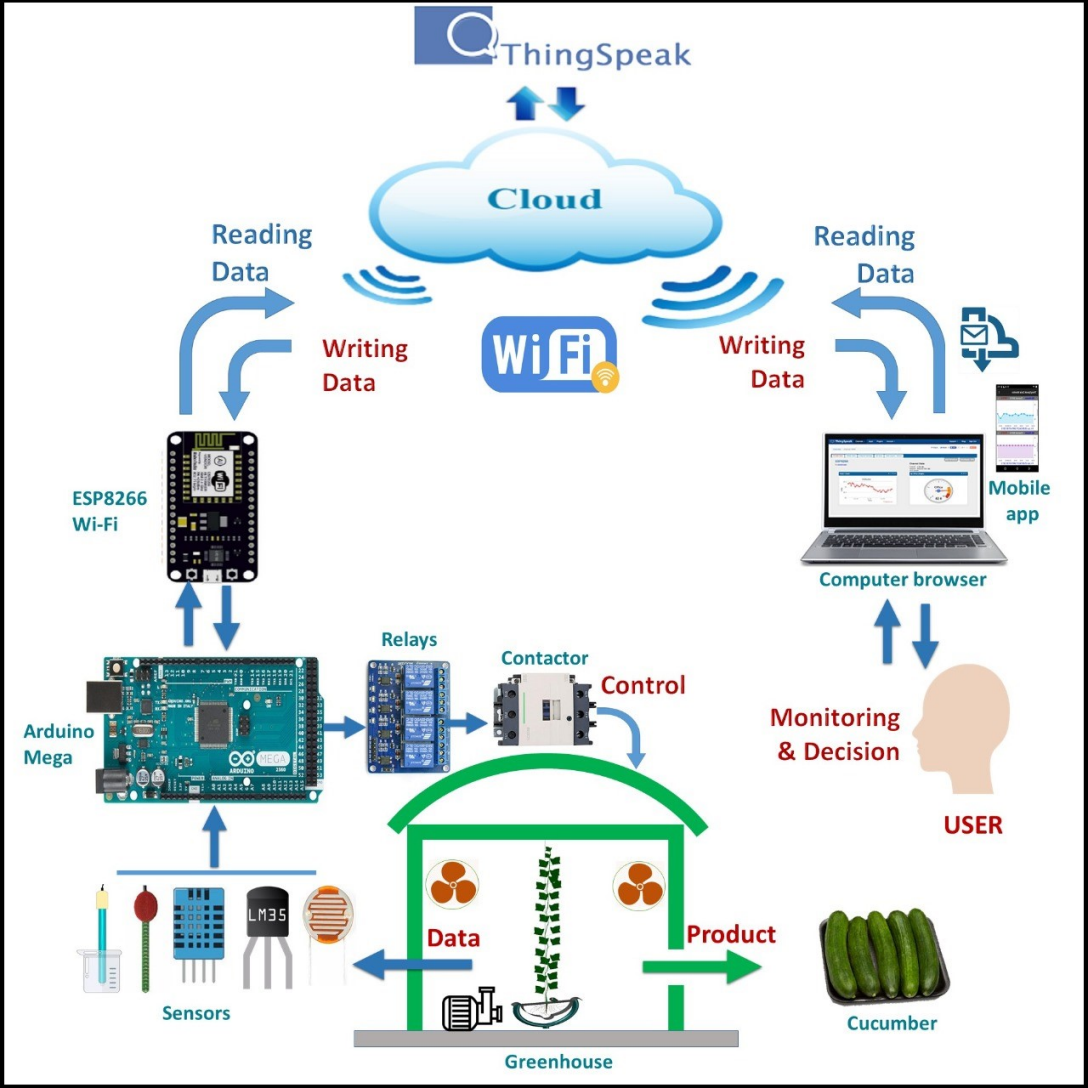
Overall system architecture of cloud-based IoT greenhouse.

The basic targets of the established cloud-based IoT platform are monitoring and controlling the greenhouse microclimate [37]. As shown in Figure 3, the established platform composed of several entities efficiently integrated together and seamlessly working to realize our targets. The main entities of the established platform are as follows:

- Arduino microcontroller,
- A set of sensors that can be connected to the Arduino microcontroller,
- A Wi-Fi module,
- A set of motors/fans,
- ThingSpeak cloud platform, and
- User interface.

The established platform starts by setting up all the sensors attached to the Arduino microcontroller. Each sensor is designated to measure a specific parameter of the greenhouse microclimate. The sensors are reporting their collected data to the Arduino microcontroller in a real-time manner. Thus, the Arduino microcontroller forwards the collected data to the Wi-Fi module which directly submits the collected data to the ThingSpeak cloud platform. Therefore, the user accesses the real-time data at any time throughout the user interface by accessing the private channel on ThingSpeak cloud platform.

Regarding the real-time decisions considered for saving the identical or semi-identical environment for the growth of our plant. There are several real-time decisions made by the Arduino microcontroller based on our software that is running on the Arduino microcontroller. Our software has the ability to control all the sensors and motors/fans attached to the Arduino microcontroller, as described in Section 2.2.3.

#### 2.2.1. Simulation Environment

**Figure 4:**
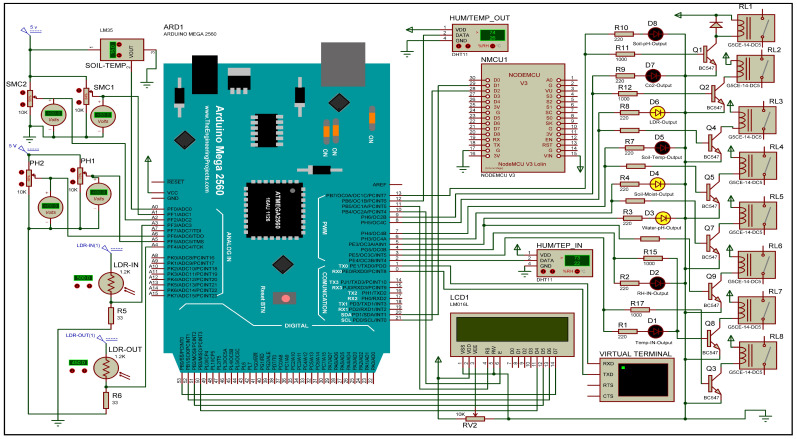
The schematic Proteus simulation model.

Referring to Figure 4, we used Proteus 8 Professional (Release 8.9 SP2 Build 28501 with advanced simulation) to conduct our simulation for all the sensors and electronic chips. This is to confirm the integrity and compatibility of these devices to operate together. Figure 4 introduces the schematic Proteus simulation model. We used an Arduino Mega 2560 microcontroller board to control our sensors and motors/fans. We used the following sensors in order to extract the required information from the greenhouse,

- DHT11 Digital Temperature & Humidity sensor,
- Analog pH sensor,
- LM35 Soil Temperature sensor,
- VH400 Soil Moisture Content sensor,
- MQ135 CO${}_{2}$ Gas sensor,
- Light Dependent Resistor (LDR)/Photoresistor,

Moreover, there are other assistant electronic devices and motors/fans were used:

- ESP8226 Highly Integrated Wi-Fi,
- 16 × 2 Character LCD,
- Virtual Terminal,
- G5CE-14-DC5 Relay,
- Us Resistors,
- Cooling Fans (CFs),
- Fogger Fans (FFs),
- Irrigation Valves (IVs),
- Irrigation Timers (ITs),
- Ventilation Fans (VFs),
- Shading Motors (SMs),
- PH electric valves (PHs),

Figure 4 shows the connections among all the aforementioned sensors and electronic devices. A detailed description for each of these devices is introduced in the following subsections.

#### 2.2.2. Hardware Design

We chose the Arduino Mega 2560 microcontroller board to control the sensors and motors/fans fixed inside the greenhouse. The Arduino Mega 2560 is based on the ATmega2560 chip. It consists of 54 digital input/output pins where 14 pins of them can be used as PWM outputs. It also has 16 analog pins for inputs, 4 UARTs (hardware serial ports), and 16 MHz crystal oscillator, which are required to support the microcontroller. At the final stage of implementation, we used 21 digital pins and 7 analog pins to interface all the required sensors and devices with the Arduino microcontroller. This will give us a great chance to improve and scale the system in future.

For the DHT11 Digital Temperature & Humidity sensor, we employed two DHT11 sensors. The first one concerned with the measurements inside the greenhouse and the second one concerned with the measurements outside the greenhouse. We considered the ambient environment because there is a significant impact on the inner humidity and temperature by the external humidity and temperature. The inner DHT11 is connected with the Arduino microcontroller through the digital pin 2. The outer DHT11 is connected with the Arduino microcontroller through the digital pin 12, as shown in Figure 4. The DHT11 sensor is equipped with a calibrated digital signal output. This sensor uses the exclusive digital-signal-acquisition technique and temperature & humidity sensing technology. Thus, it ensures high reliability and excellent long-term stability. Based on the obtained measurements from the DHT11 sensor, our platform will control the cooling fans and fogger fans inside the greenhouse.

For the analog pH sensor, we have used two pH sensors for measuring the pH for the water at the water tank, and before feeding the greenhouse lines. The task of our system is to stabilize the pH level at 7 within a very short time based on the quantity of water needed. The pH sensor of the water tank is connected to the Arduino microcontroller at pin A6 and the greenhouse pH sensor is connected to the Arduino microcontroller at pin A5, as shown in Figure 4. It should be mentioned that the measurements are taken as real-time measurements. Based on the measurements obtained from the analog pH sensor, our platform will control the PH electric valves to stabilize the water pH level.

The LM35 Soil Temperature sensor is a precision integrated-circuit device for measuring the temperature. Its output is the voltage that linearly-proportional to the Centigrade temperature. It is connected with the Arduino microcontroller through pin A0, as shown in Figure 4. We used this sensor for measuring the soil temperature in our system. This sensor has a very useful feature over other linear temperature sensors calibrated in Kelvin, where LM35 eliminates the user’s interaction to subtract a large constant voltage from the output to obtain the appropriate Centigrade scaling. Also, there is no need for any external trimming or calibration to provide typical accuracies.

The VH400 soil moisture content sensor is a low-cost professional electronic sensor. We employed two VH400 sensors, they are connected to the Arduino microcontroller through pin A1 and A2, as shown in Figure 4. We carefully chose this sensor because of its multiple advantages. It owns high sensitivity, and waterproof and rugged. Also, it has the ability to ignore the soil’s salt. Since, it is very thin, the probe does not disturb roots. Moreover, it is extremely fast, thus it suits our real-time measurements. The output voltage is by default proportional to the soil’s moisture level. It is responsible for stopping watering when the soil is wet in our greenhouse. Based on the measurements obtained from the VH400 sensor, our platform will control the irrigation valves.

The MQ135 CO${}_{2}$ gas sensor is a perfect air quality sensor, therefore, we used one unit inside the greenhouse to explore the air quality. We interfaced the MQ135 with the Arduino microcontroller through digital pin 13. This sensor is not shown on the schematic simulation model, because its library was not found for the Proteus simulator. Indeed, we have specifically chosen this sensor because it displays its output as analog voltage 0–5 V or as digital output. In our system, we adopted the the digital output format, because its sensitivity can be varied using the potentiometer. It is enjoying with a wide detection scope with extremely fast performance. Based on the measurements obtained from the MQ135 sensor, our platform will control the ventilation fans. The two LDRs are interfaced with the Arduino microcontroller through pin A3 and A4. Based on the measurements obtained from the inner LDR sensor, our platform will control the shading motors in the greenhouse.

The ESP8226 is a highly integrated Wi-Fi module, we interfaced it with the Arduino microcontroller through two digital pins (20 and 21). The ESP8226 is equipped with self-contained Wi-Fi networking capabilities. This module has a high-speed cache, which helps in increasing the system performance as well as optimizing the memory. It is an optimal solution for energy saving because its VDDA pin accepts Analog Power 2.5 V:3.6 V. Moreover, it quickly switches between wake-up and sleep mode for efficient energy-saving purposes. In our platform, ESP8226 is responsible for internet connectivity. As soon as the information is collected from our sensors, it is sent to the ESP8226 as a string data type. Then, this string is sent to our private channel on the ThingSpeak platform [38].

The 16×2 character LCD is an electronic LCD device uses liquid crystal to produce a visible image. It has the ability to display 16 characters per line in two of such lines. It is interfaced with the Arduino microcontroller through six digital pins (10, 11, 50, 51, 52, and 53). It is used in our platform to display some basic information such as starting up the system, starting up the sensors, fixing a specific parameter, sending data to the cloud, and so on. The G5CE-14-DC5 Relay is ideal for switching power in household appliances or for outputs from industrial devices. We employed eight G5CE-14-DC5 relays in our system. Each relay is concerned with the output for a specific sensor and connected to the related motors/fans inside the greenhouse.

#### 2.2.3. Software Design

We considered the following basic aims before designing the software of our system:

- It is readable by embedding the required comments for each step,
- It displays real-time information about the status of the system at every time, such as “Starting up the System”, “Starting up the Sensors”, “Fixing a Specific Measure (pH, Moisture, Temperature, and so on)”, “Sending Data to Cloud”, and so on.
- It has the ability to alert the user if any up-normal issues occurred, and
- It has sufficient flexibility to be extended by inserting more measurements.

**Figure 5:**
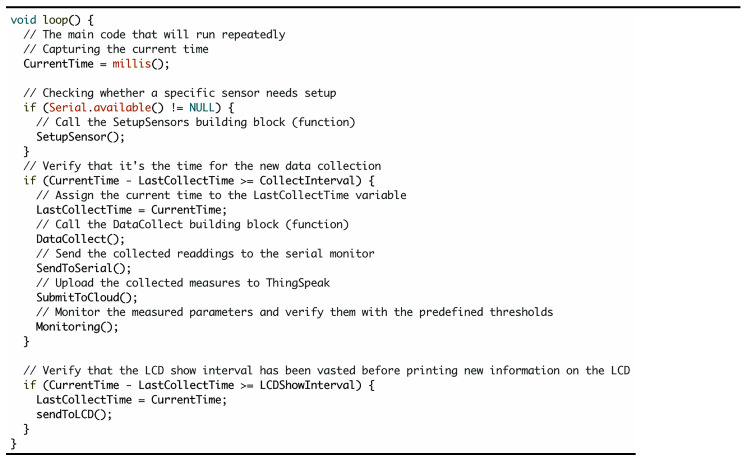
The main code to be repeated in our system.

We carefully followed the formal system life cycle to obtain an efficient software design by satisfying the following basic requirements,

- Requirements: According to our real-time field experiments, we defined the required information to be collected from the greenhouse as well as the desired results from our software.
- Analysis: We broke down the main system software into manageable building blocks. As shown in Figure 5, our system software is represented in six basic building blocks:
  - SetupSensors( )
  - DataCollect( )
  - SendToSerial( )
  - SubmitToCloud( )
  - Monitoring( )
  - SendToLCD( )
- Design: We decided the required data objects and operations.
- Refinement and Coding: We implemented the required algorithms and data objects.
- Verification: We tested and introduced the correctness proofs for our systems measurements and decisions based on parallel real field measurements.

The detailed description of our automated system’s software manageable building blocks is as follows:

- SetupSensors( ):This building block is very important to communicate with most of the system sensors for various issues, such as resets, manual readings, and calibration. We used the SetupSensors( ) function for this purpose, where each sensor in our system is identified by a unique ID. Once the user passes the ID of the sensors to be adjusted for the SetupSensors( ) function, a new getCommand( ) function is instantiated to receive the user commands and submit them to the sensor for further adjustment.
- DataCollect( ): At each data collection interval, the DataCollect( ) function grab all the measurements and readings from the nine sensors employed in our platform. It should be mentioned that each sensor uses its own library to complete its task based on its own functionality.
- SendToSerial( ): This function is responsible for printing real-time measurements obtained from each sensor in the platform on our serial terminal.
- SubmitToCloud( ): As soon as the data has been collected using the DataCollect( ) function, this function gathers all the data in a variable of type string called “CollectedData” and sends it to the ESP8226. Once the ESP8226 receives this string variable, it submits the collected data to ThingSpeak. It should be mentioned that this process is executed once at each data collection interval.
- Monitoring( ): This function monitors the measured parameters. It also controls the system motors/fans based on the predetermined thresholds for each parameter. Moreover, this function keeps the lights concerning a specific parameter on while adjusting its value to be within the normal and accepted levels. For example, if the greenhouse temperature became greater than 26 ${}^{{}^{\circ}}$C, then the temperature alarm light will on and the cooling fans will be activated to bring the greenhouse temperature back to be 24 ${}^{{}^{\circ}}$C. Then, the cooling fans and the temperature alarm light will stop working when the greenhouse temperature become 24${\;}^{{}^{\circ}}$C. It should be mentioned that the system monitoring state is normally viewed on our serial monitor and LCD.
- SendToLCD( ): One of our main objectives is to keep track for up to date information about all the system parameters. Thus, this function is used to scroll the information on the LCD every five seconds. It is worth to mention that, the information that is going to be displayed on the LCD is the alarming situations only such as fixing pH, fixing Temp, fixing humidity, and so on.

### 2.3. Substrate-Soilless Culture

In the substrate-soilless cultivation systems, the solid medium works as a support for the plant and provides the plant root with the needed oxygen [39]. For the cucumber cropping in this research, we used a mixture of peat moss and perlite (1:1) as recommended by Mazahreh et al. [40]. In general, the peat moss medium consists of a plethora of organic elements widely available in several regions in the world. It is widely used in the scope of many reasons including relative low costs and weight, low distinguished high water-holding capacity, free from pests and diseases [41].

The cucumber plant (Al Gawhara F1), as a recommended cultivar by the greenhouse supervisor, was planted in the growing medium with a plant density of 3.5 plant/m${}^{2}$ [42]. The cucumber seeds were planted directly in the growing medium on 15 August 2020. During the first week, all plants were daily irrigated with water only. After that, the nutrient solution was added to the irrigation water during the plant growth using a Papadopolus formula with fertigation method [43]. The fertilizer solutions were prepared in a separate tank and are added to the irrigation system as needed using the automatic control system instantiated in the greenhouse. The nitric acid solution was used to maintain the pH value between 5.5 and 6.

For each cultivation line inside the greenhouse, a double row of drip tubing with a distance between two droppers of 40 cm was installed to assign one emitter for each plant where the final distance between plants is 20 cm. The size of the drip tubing was 16 mm and the dripper discharge was 2000 cm${}^{3}$/h. The water pressure was controlled in the irrigation tube at 200 kPa using pressure regulators. Based on the schedule of daily irrigation, the solenoid valves (24 Volt) controlled by the digital timer (Model: TM919, HHT, 24 h, 7 days, Guangdong, China) are utilized to control the water supply. Using a digital flow meter, the cumulative applied irrigation water through the experiment is potentially observed.

The treatments were arranged in a randomized complete block design (RCBD) with two different irrigation control methods (daily using a digital timer and automatic control using volumetric soil moisture sensor) in 10 replicates. The plants under the timer control method are irrigated two times per day from the first week until the end of the experiment based on the stage of the plant growth. The plants under the volumetric soil moisture control system were irrigated automatically based on the medium moisture content. During the system implementation, the temperature, relative humidity, and pest control were similar for the two irrigation control systems. The digital water flow meters were connected to measure the actual water delivered to each irrigation control system.

### 2.4. Measurements

#### 2.4.1. Cucumber Fruit Characteristics

In order to realize the impact of the established platform on the cucumber fruit growth, we investigated the fruit attributes with/without our platform. The following characteristics for cucumbers fruit were investigated:

- Dimensions, projected area, and fruit weight:
  We obtained the cucumber projected area, weight and dimensions using a digital camera; model Nikon Coolpix B600, camera with a LED light source. The gathered cucumber images are processed using the free open source Fiji-ImageJ2 [44] to identify the desired weight and dimensions, where the former is estimated using the sartorius electronic balance [45].
- Volume:
  The cucumber volume is determined according to the gas-displacement methodology in isothermal systems at pressure and temperature of 200 kPa and 25 °C, respectively, using the low-pressure cylinder and the treatment chamber. The volume of the fruit sample was estimated using the following formulas [46]:
  (1) $$\begin{array}{c} P_{cy}\times V_{cy}=P_{eq}V_{cy}+P_{eq}\left(V_{ch}-V_{sa}\right) \end{array}$$
  (2) $$\begin{array}{c} V_{sa}=V_{ch}-V_{cy}\left(\left(P_{cy}-P_{eq}\right)⁄P_{eq}\right) \end{array}$$
  where $P_{cy}$ (in kPa) and $V_{cy}$ (in cm${}^{3}$) are the pressure and volume of the low-pressure cylinder, $P_{eq}$ (in kPa) is the equilibrium-pressure, $V_{sa}$ (in cm${}^{3}$) is the sample volume, and $V_{ch}$ (in cm${}^{3}$) is the treatment chamber.
- Density:
  The cucumber density is estimated by the ratio of the sample weight to its volume, which estimated by the gas and water displacements.
- Color:
  The cucumber fruit color was identified using the color spectrophotometer that developed by Hunter Laboratory. This step is performed based on the color system of chromaticity coordinates a (- a = greenness, a = redness), lightness factor L (0 = black, 100 = white), the chromaticity coordinates a (- a = greenness, a = redness), and b (- b = blueness, b = yellowness) as described in [47]. For the experiments of this paper, the color parameters are measured before and after treatment using 100 cucumber samples randomly selected. In this case, we calculate the chroma (*C*) and hue angle (*h*) as given in [47]:
  (3) $$\begin{array}{ccc} C & = & \sqrt{(}a^{2}+b^{2}) \end{array}$$
  (4) $$\begin{array}{ccc} h & = & arctan(b/a) \end{array}$$
- Moisture content:
  The fruit moisture content is estimated through dehydrating a sample in a vacuum-drying oven (Model: LVO-2041P) at about 70 °C following the common analysis methods [48]. The fruit samples were randomly selected from the cultivation lines of cucumber in the greenhouse under study, such that each fruit sample is used to determine the physicochemical parameters. Whereas, the width and length of each samples is estimated using any suitable tool, such as digital vernier slide caliper.
- pH:
  The pH of the cucumber fruit was determined based on the standard analysis methods (AOAC) using the pH meter (Model: EC500, Extech, China) [48].
- Total soluble solids and fruit firmness:
  The cucumber total soluble solids and firmness are specifically estimated using the Koehler penetrometer (Thomas Scientific, Swedesboro, NJ, USA) and laboratory refractometer (Model: RFM 840, Richmond Scientific Ltd. Unit 9, Lancashire, UK), respectively.
- Hardness:
  To measure the cucumber hardness we selected 10 samples randomly selected from each treatment. Usually, the hardness of any fruit crops is measured using the maximum pressure (N/cm${}^{2}$) required to penetrate the body of the sample using a fruit pressure tester (Model: FT 327), which is fitted with a stainless steel cylindrical probe with a diameter of 8 mm.

#### 2.4.2. Irrigation Water Requirement

In our experiments, the irrigation water reached the surface area of the growing media from the irrigation emitter and then infiltrate into the media itself [41]. The infiltrated water amount is divided into various parts **(i)** The evaporated amount directly from the surface of growing media, **(ii)** The accumulated amount within the plant root zone, **(iii)** The amount that taken up by the plant required for transpiration and growth, **(iv)** The drained amount that downward beyond the plant root zone. The water balance in the plant is based on mass conservation, which states that change in growing medium moisture content of a plant root zone is equal to the difference between the amount of irrigation water added, and the amount of water withdrawn in a given time interval. This can be represented mathematically as follows:

(5) $$\begin{array}{c} \Delta S=Q_{in}-Q_{out}/\Delta_{t}S \end{array}$$

where ΔS is the difference in the moisture storage in the root zone medium, $Q_{in}$ is the amount of irrigation water added (m${}^{3}$), $Q_{out}$ is the amount of water withdrawn (m${}^{3}$). The reference value of evapotranspiration for the controlled environment of the greenhouse is determined using the methodology proposed by FAO based on the Penman-Monteith model [49]. The crop evapotranspiration of the crop is determined as follows:

(6) $$\begin{array}{c} ET=K_{c}-ET_{o}=I-R-D-\Delta S \end{array}$$

where, *ET* (mm) refers to the crop evapotranspiration, $K_{c}$ refers to the crop’s coefficient, I refers to the amount of irrigation water applied (mm), *R* refers to the amount of runoff (mm), *D* refers to the amount of water percolation (mm), and Δ*S* is given in Equation (5). In this case, the irrigation water amount is controlled, so the deep percolation and runoff are considered to be equal to zero, and the $K_{c}$ in the range between 0.8 to 1.6. The following requirements are calculated as follows,

- Amount of irrigation water:
  The measurement of the required irrigation water is conducted by using the digital flow meter whereas the drainage water volume is estimated in the collection tank. The plant water uptake is estimated based on the water balance by measuring the irrigation water input and the total of various outputs. The cumulative applied irrigation water during the experiment life time is estimated using the digital flow meter (Model: K24, SUNNY, Linyi, China). The amount of daily irrigation water requirement is calculated as follows:
  (7) $$\begin{array}{c} IWR=A_{s}\times ET \end{array}$$
  where, IWR refers to the irrigation water requirement (m${}^{3}$/day), $A_{s}$ refers to the target surface area (m${}^{2}$), *ET* (mm) refers to the crop evapotranspiration as given in Equation (6).
- Meteorological data:
  The meteorological data of the greenhouse is including the relative humidity (%), the minimum and maximum air temperature (°C), the solar radiation (W/m${}^{2}$), sunshine duration (h), wind speed (km/h) are recorded by a weather station installed at the greenhouse.
- Growth medium moisture content:
  In this research, the amount of irrigated water was controlled based on the growing medium moisture content, which is estimated by using a handheld moisture meter (Model: VG-Meter-200, Vegetronix, UT, USA). The real-time moisture content is recorded by using a waterproof moisture sensor probe (Model: VH400 Vegetronix, UT, USA). To avoid any bias, the moisture content probe is calibrated versus the real medium water content that determined the gravimetrically at the greenhouse site.

#### 2.4.3. Plant Parameters

The cucumber chlorophyll content is estimated using a chlorophyll meter (Model: SPAD 502, Konica–Minolta, Osaka, Japan) at the same time intervals. Moreover, the plant temperature is determined using a thermal imaging camera (Model: FLIR T335, Danderyd, Sweden).

#### 2.4.4. Water Use Efficiency of Cucumber

The water use efficiency (*WUE*) of the cucumber was estimated using the following form:

(8) $$\begin{array}{c} WUE=Y/WA \end{array}$$

where *WUE* is given in (kg/m${}^{3}$), *Y* is the total marketable yield in (kg), *WA* is the amount of applied irrigation water used in (m${}^{3}$).

#### 2.4.5. Electrical Energy

The total electrical energy consumed to operate the greenhouse was calculated using the following formula,

(9) $$\begin{array}{c} E=P_{t}\times \;T \end{array}$$

(10) $$\begin{array}{c} P_{t}=P_{\mathrm{ph}1}+P_{\mathrm{ph}2}+P_{\mathrm{ph}3} \end{array}$$

(11) $$\begin{array}{c} P_{\mathrm{ph}}=V_{\mathrm{ph}}\times I_{\mathrm{ph}}\times \mathrm{PF} \end{array}$$

where *E* is the electrical energy (Watt/h), $P_{t}$ is the total three-phase power (Watt), and *t* is the target time (h), $P_{ph}$, $V_{ph}$, $I_{ph}$ are the power (W), voltage (V), and current (A) of the three phases (ph), and *PF* is the power factor, which usually equals to 0.95.

### 2.5. Statistical Analysis

All the aforementioned measurements including the cucumber fruit characteristics and the water use efficiency, are analyzed using the one-way analysis of variance ANOVA using the statistical analysis program of IBM SPSS version 23 (SPSS Inc., Chicago, IL, USA).

## 3. System Implementation and Results

### 3.1. Cloud-Based IoT Monitoring

**Figure 6:**
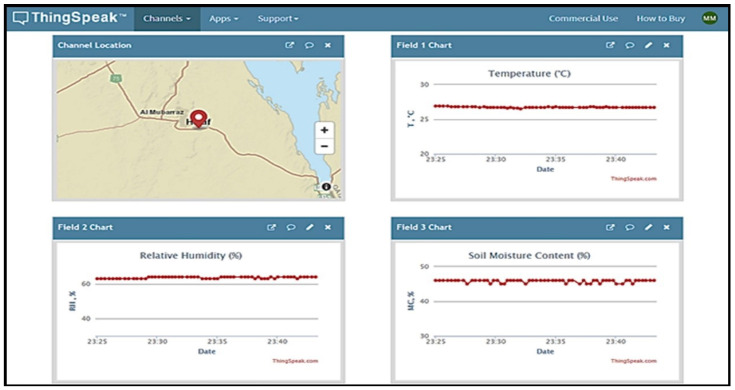
A screenshot of the received microclimate measurements for humidity, temperature, and soil moisture content, via the ThingSpeak platform.

As we explained in the previous sections, the established cloud-based IoT platform is an automated system for monitoring and controlling the microclimate and actuators inside the greenhouse. Therefore, based on the sensors’ measurements, the actuators will turn either on or off. Moreover, all real-time measurements of the greenhouse microclimate conditions will be sent to the open-source “ThingSpeak” platform [38] for further process. Figure 6 shows the real-time measurement results on a window after linking to the ThingSpeak platform and selecting a private channel to monitor the microclimate parameters. The microclimate data are also stored in the cloud as Google spreadsheets, which were used for visualization and analysis of the microclimate parameters. Using this cloud-based platform, the user remotely monitors the greenhouse and accesses the relevant data of the greenhouse microclimate to decide the suitable action based on the current situation.

### 3.2. Greenhouse Microclimate Control and Automation

**Figure 7:**
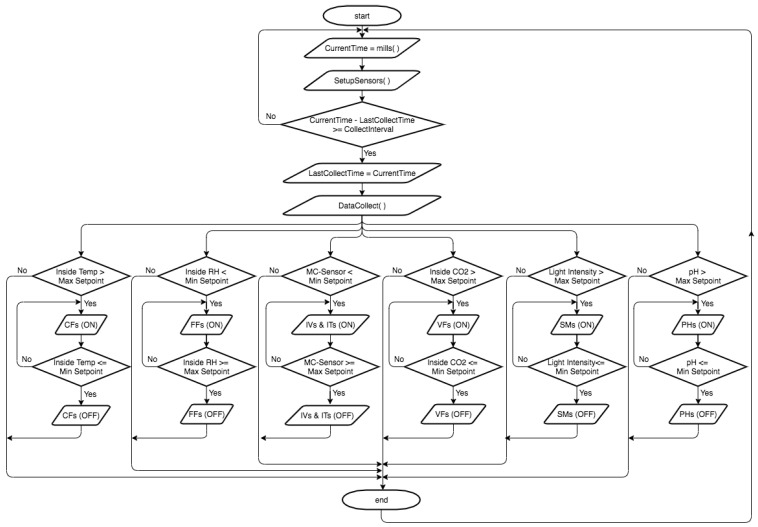
The control structure flowchart for the established cloud-based IoT platform.

**Figure 8:**
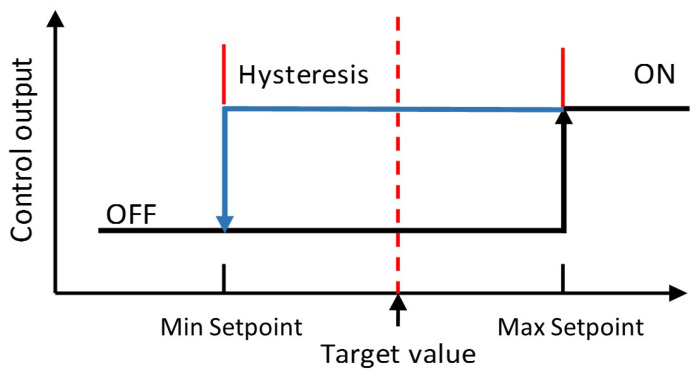
A schematic showing hysteresis between ON and OFF operations.

This section introduces the main results for controlling and automating parameter of interest according to our system software, as shown in Figure 7. The figure shows the control structure for six parameters. The ON/OFF control action of the relays and contactors turns the output ON or OFF based on the set point of the considered parameter. The output frequently changes according to the parameter value changes. These changes shorten the life of the relays and contactors or unfavorably affect the valves, fans, and motors of pumps connected to the controller. To prevent this problem from occurring, a hysteresis band was created between the ON and OFF operations to protect the relays and actuators. As shown in Figure 8, the control of each parameter was achieved based on the hysteresis between the target value and setpoint by checking its value twice one for the max setpoint and the second one for the min setpoint.

**Figure 9:**
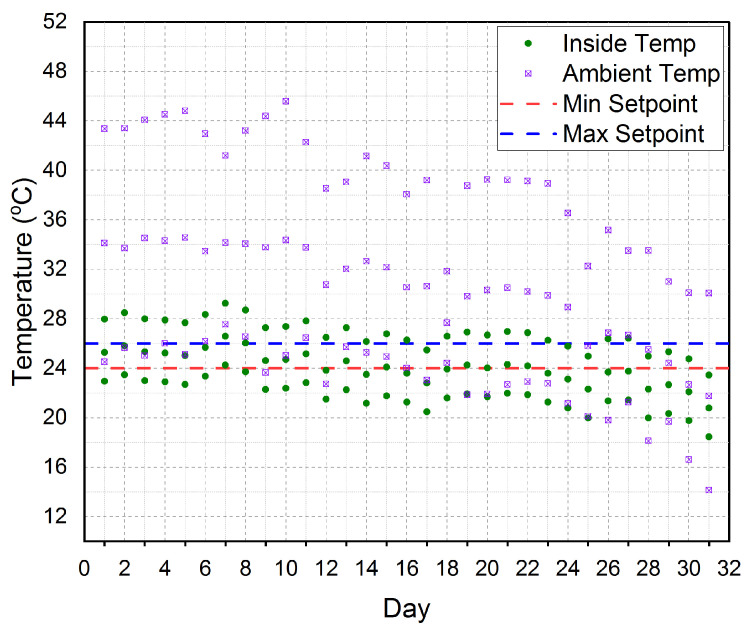
The three months average for inner greenhouse temperature (Inside Temp) and outside temperature (Ambient Temp) according to the minimum setpoint (Min Setpoint) and maximum setpoint (Max Setpoint) considered in our platform.

The established cloud-based IoT platform was automatically controlling the greenhouse inner temperature and the results for a period of three months are shown in Figure 9. The figure shows the three months average measured inner temperature (InsideTemp) and the three months average measured outside greenhouse temperature (AmbientTemp). The figure also shows the minimum setpoint (MinSetpoint) and the maximum setpoint (MaxSetpoint) for the inner greenhouse temperature.It is clear that the ambient temperature impacts the inner greenhouse temperature.

Thus, we should have a significant tool to control the inner greenhouse temperature. According to our real-time experiments and our software, the $Min\;Setpoint=24{\;}^{{}^{\circ}}$C and the $Max\;Setpoint=26{\;}^{{}^{\circ}}$C. These setpoints are considered as recommended for cucumber cultivation in [50]. Thus, our target temperature inside the greenhouse was fixed at 25 ${}^{{}^{\circ}}$C. Our platform automatically controls the inner greenhouse temperature by controlling the Cooling Fans (CFs) on/off. Once the inner greenhouse temperature became greater than MaxSetpoint$(26{\;}^{{}^{\circ}}$C), the CFs will be turned on to bring the inner greenhouse temperature back to be equal to the MinSetpoint$(24{\;}^{{}^{\circ}}$C). Then, the CFs will be turned off. Therefore, as shown in the figure the inner greenhouse temperature is tightly scattered around the MinSetpoint and the MaxSetpoint.

**Figure 10:**
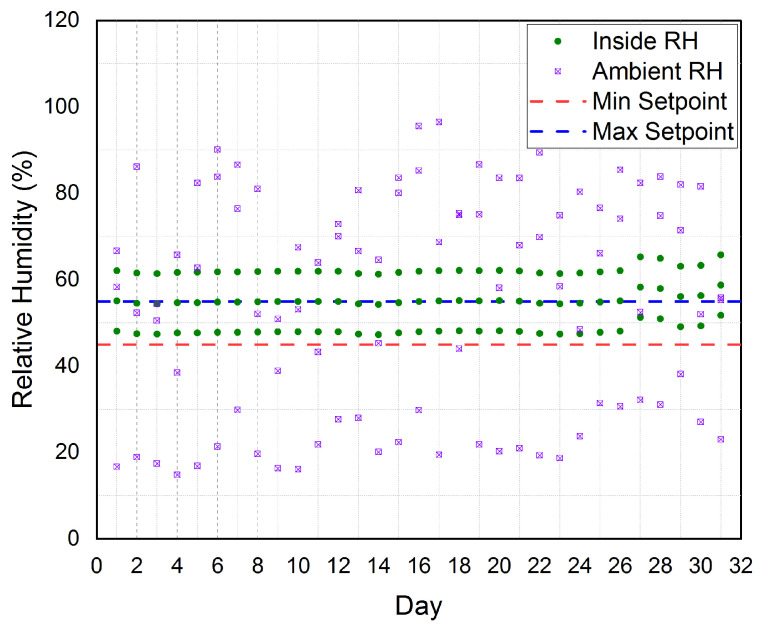
The three months average for inner greenhouse relative humidity (Inside RH) and outside relative humidity (Ambient RH) according to the minimum setpoint (Min Setpoint) and maximum setpoint (Max Setpoint) considered in our platform.

Based on the aforementioned discussion of Figure 9, we conclude that the established cloud-based IoT platform was successfully controlled the inner greenhouse temperature to be very near to the target temperature inside the greenhouse $(25{\;}^{{}^{\circ}}$C). According to the Relative Humidity (RH), the established cloud-based IoT platform is automatically controlling the inner greenhouse relative humidity and the results for a period of three months are shown in Figure 10. The figure shows the three months average measured inner relative humidity (InsideRH) and the three months average measured outside greenhouse relative humidity (AmbientRH). The figure also shows the minimum setpoint (MinSetpoint) and the maximum setpoint (MaxSetpoint) for the inner greenhouse relative humidity. It is clear that the outside relative humidity impacts the inner greenhouse relative humidity. Thus, we should have a significant tool to control the inner greenhouse relative humidity. According to our real-time experiments and our software, the MinSetpoint=45% and the MaxSetpoint=55%. Thus, our target relative humidity inside the greenhouse was 50%.

The established platform automatically controls the inner greenhouse relative humidity by controlling the Fogger Fans (FFs) on/off. Once the inner greenhouse relative humidity became smaller than MinSetpoint(45%), the FFs will be turned on to raise the inner greenhouse relative humidity to be greater than or equal to the MaxSetpoint(55%). Then, the FFs will be turned off. Therefore as shown in the figure, the inner greenhouse relative humidity is tightly scattered around the MinSetpoint and the MaxSetpoint. According to the aforementioned discussion of Figure 10, we conclude that the established cloud-based IoT platform has successfully controlled the inner greenhouse relative humidity to be very near to our target relative humidity inside the greenhouse, i.e., 50%.

**Figure 11:**
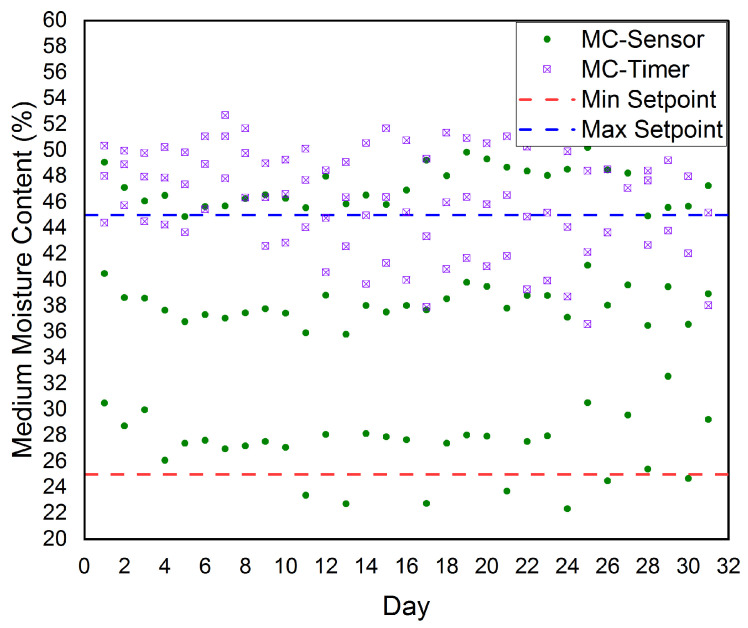
The three months average for the sensed growth medium moisture content (MC-Sensor) and irrigation timer (MC-Timer) according to the minimum setpoint (Min Setpoint) and maximum setpoint (Max Setpoint) considered in our platform.

The growth medium moisture content (MC) in our platform was measured using two different methods, the first one was using the sensed MC (MC–Sensor) and the second method was using an irrigation timer (MC–Timer). The established cloud-based IoT platform is automatically controlling both the MC–Sensor and the MC–Timer, where the results for a period of three months are shown in Figure 11. The figure shows the three months average measured MC–Sensor and the three months average measured MC–Timer. The figure also shows the minimum setpoint (MinSetpoint) and the maximum setpoint (MaxSetpoint) for both MC–Sensor and MC–Timer. According to our real-time experiments and our software, the MinSetpoint=25% and the MaxSetpoint=45%. Thus, our target growth medium moisture content was 35%.

Our platform automatically controls the growth medium moisture content by controlling the Irrigation Valves (IVs) on/off for the MC–Sensor. Also, our platform automatically controls the growth medium moisture content by controlling the Irrigation Timers (ITs) on/off for the MC–Timer. Once the growth medium moisture content became smaller than MinSetpoint(25%), the IVs and ITs will be turned on to raise the growth medium moisture content to be greater than or equal to the MaxSetpoint(45%). Then, the IVs and ITs will be turned off. Therefore as shown in the figure, the growth medium moisture content is tightly scattered around the MinSetpoint and the MaxSetpoint. According to the aforementioned discussion of Figure 11, we conclude that the established cloud-based IoT platform has successfully controlled the soil moisture content to be very near to our target soil moisture content, i.e., 35%.

**Figure 12:**
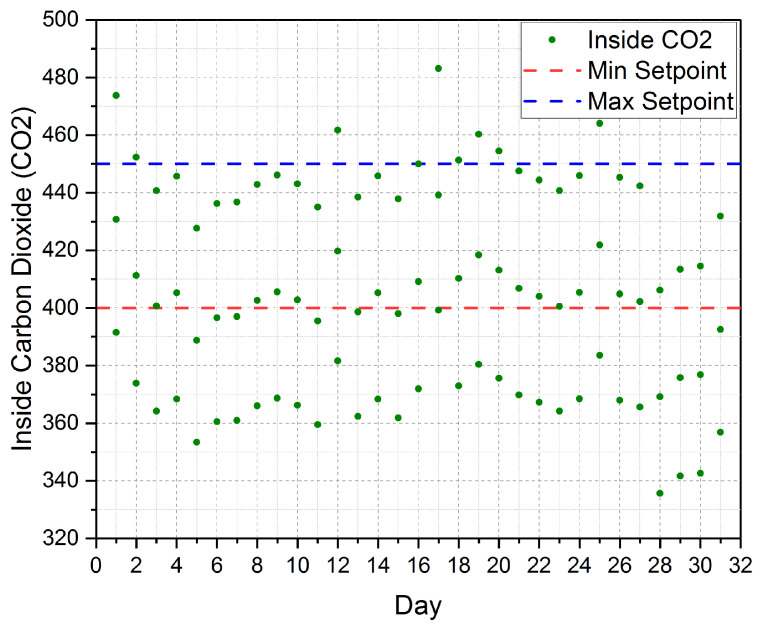
The three months average for inner carbon dioxide (Inside CO${}_{2}$) according to the minimum (Min Setpoint) setpoint and maximum setpoint (Max Setpoint) considered in our platform.

The established cloud-based IoT platform is automatically controlling the inner greenhouse Carbon Dioxide (CO_2_) for a period of three months is shown in Figure 12. The figure shows the three months average measured $Inside\;{\mathrm{CO}}_{2}$. The figure also shows the minimum setpoint (MinSetpoint) and the maximum setpoint (MaxSetpoint) for the inner carbon dioxide. According to our real-time experiments and our software, the MinSetpoint=400 and the MaxSetpoint=450. Thus, our inner target carbon dioxide is 425. Our platform automatically controls the inner carbon dioxide by controlling the Ventilation Fans (VFs) on/off. Once the inner carbon dioxide became greater than MaxSetpoint(450), the VFs will be turned on to bring the inner carbon dioxide back to be equal to the MinSetpoint(400). Then, the VFs will be turned off. Therefore, as shown in the figure the inner carbon dioxide is tightly scattered around the MinSetpoint and the MaxSetpoint. According to the aforementioned discussion of Figure 12, we conclude that the established cloud-based IoT platform has successfully controlled the inner carbon dioxide to be very near to our target CO${}_{2}$ inside the greenhouse, i.e., 425.

**Figure 13:**
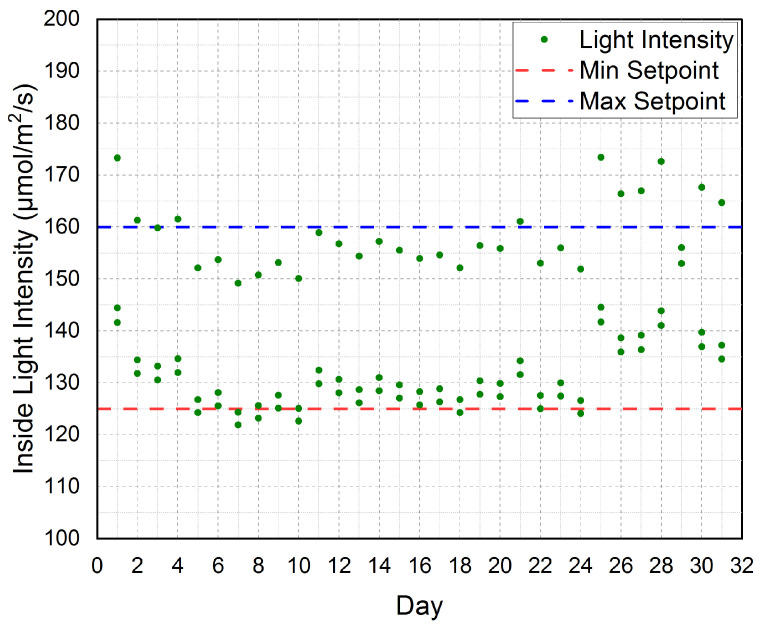
The three months average for inner light intensity (Light Intensity) according to the minimum setpoint (Min Setpoint) and maximum setpoint (Max Setpoint) considered in our platform.

The established cloud-based IoT platform is automatically controlling the inner light intensity for a period of three months is shown in Figure 13. The figure shows the three months average measured light intensity. The figure also shows the minimum setpoint (MinSetpoint) and the maximum setpoint (MaxSetpoint) for the inner light intensity. Since the plants use light energy between 400 to 700 nanometers, the area is described as Photosynthetically Active Radiation (PAR). PAR is based on the photons number in a certain waveband incident per unit time (s) on a unit area (m${}^{2}$) divided by the Avogadro constant that equals 6.022 × 1023/mol. A conversion value of 0.0185 was used to convert the Lux to μmol/m${}^{2}$/s at the PAR area as described in [51,52].

According to our real-time experiments and our software, the Min Setpoint = 125 μmol/m${}^{2}$/s and the Max Setpoint = 160 μmol/m${}^{2}$/s. Thus, our inner target light intensity was 145μmol/m${}^{2}$/s. Our platform automatically controls the inner light intensity by controlling the Shading Motors (SMs) on/off. Once the inner light intensity became greater than MaxSetpoint(160μmol/m${}^{2}$/s), the SMs will be turned on to bring the inner light intensity back to be equal to the MinSetpoint (125 μmol/m${}^{2}$/s). Then, the SMs will be turned off. Therefore, as shown in the figure the inner light intensity is tightly scattered around the MinSetpoint and the MaxSetpoint. According to the aforementioned discussion of Figure 12, we conclude that the established cloud-based IoT platform has successfully controlled the inner light intensity to be very near to our target light intensity inside the greenhouse, i.e., 145 μmol/m${}^{2}$/s.

**Figure 14:**
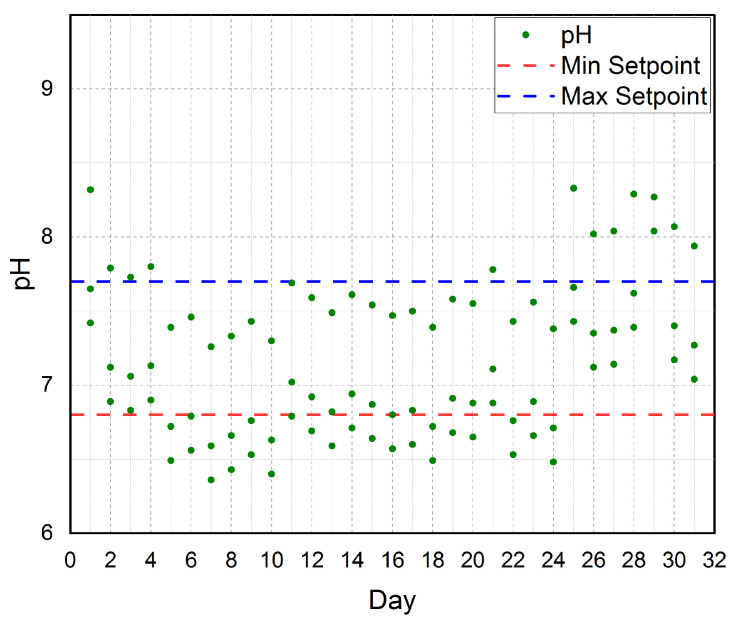
The three months average for water pH according to the minimum setpoint and maximum setpoint considered in our platform.

The established cloud-based IoT platform is automatically controlling the water pH for a period of three months as shown in Figure 14. The figure also shows the minimum setpoint (MinSetpoint) and the maximum setpoint (MaxSetpoint) for the water pH. According to our real-time experiments and our software, the MinSetpoint=6.8 and the MaxSetpoint=7.7. Thus, our water pH is 7.25. Our platform automatically controls the water pH by controlling the PH electric valves (PHs) on/off. Once the water pH became greater than MaxSetpoint(7.7), the PHs will be turned on to bring the water pH back to be equal to the MinSetpoint(6.8). Then, the PHs will be turned off. Therefore, as shown in the figure the water pH is tightly scattered around the MinSetpoint and the MaxSetpoint. According to the aforementioned discussion of Figure 14, we conclude that the established cloud-based IoT platform has successfully controlled the water pH to be very near to our target water pH (7.25).

**Figure 15:**
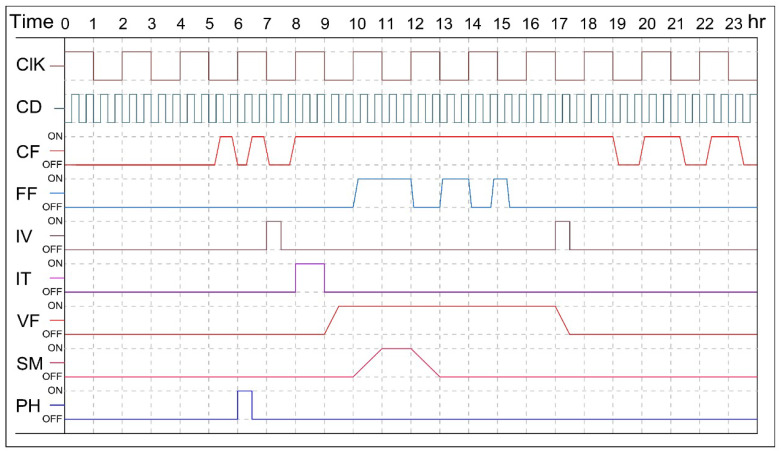
Timing diagram of ON/OFF operations of the greenhouse actuators. Clk: Clock for timing; CD: Collected Data; CF: Cooling Fan; FF:Fogger Fan; IV: Irrigation Valve controlled by the moisture sensors; IT: Irrigation valve controlled by the digital Timer; VF—Ventilation Fan.

Figure 15 shows the timing diagram of the time-interval in which collected data was sent from the Wi-Fi module to the ThingSpeak platform. In addition, the figure shows other properties including the turning (on/off) operations of the cooling fans based on the temperature setpoints, the fogger fans based on the relative humidity setpoints, the ventilation fans based on the set points of CO${}_{2}$, irrigation valve controlled by the moisture sensors based on the medium moisture content set points, and irrigation valve based on the set points of the timer.

### 3.3. Impact on Cucumber Characteristics

Results in Table 1 show the irrigation water applied using the moisture sensor control resulted in significantly high values concerning cucumber fruit length, width, weight, and total soluble solids (TSS). Simultaneously, irrigation control using irrigation timing led to an increase in the diameter of the cucumber, aspect ratio, hue angle, Chroma, and moisture content. There are no significant differences among the average values of the cucumber fruit projected area, volume, density, lightness, hardness, and pH when using moisture sensing or digital timer method for irrigation under the application of IoT system. Generally, the results indicate that the irrigation control system using the medium moisture sensor improved the cucumber quality parameters regarding its color and appearance. This finding is due to maintaining the medium moisture content level within the cucumber root zone with a perfect dose.

Table 2 shows the impact of irrigation water applied by the medium moisture control and irrigation timing control on the plant characteristics. The results showed that the irrigation control using the medium moisture significantly raised the averages of chlorophyll and the number of fruit, and the total yield per plant. At the same time, the irrigation control by the timing system led to a significant increase in the average plant temperature. This result is due to a delay in the water supply to the plant, which leads to a rise in plant temperature.

**Figure 16:**
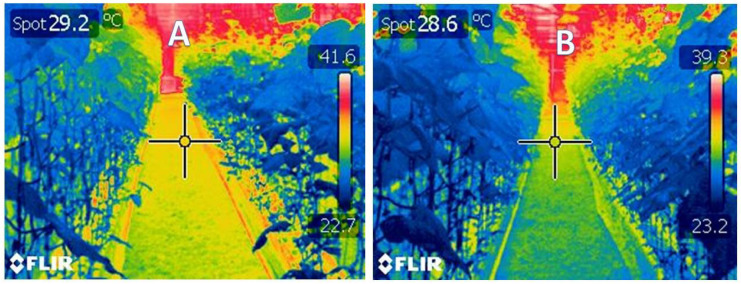
Thermal image of plant inside the greenhouse. (**A**) plant irrigated using digital timer control system (**B**) plant irrigated using growing medium moisture sensor. Both images were taken inside the same greenhouse at the same time (11.00 AM). The image B shows a lower temperature using the growing medium moisture sensor, due to the regularity of the irrigation water.

Figure 16 shows two thermal images of the temperature distribution inside the greenhouse either with normal control system (image A) or with the established control system (image B). It is clear that the blue color dominates the image B dues to the temperature degrees on image B are lower than those of image A. In other words, the established platform successfully maintained the plant temperature within the ideal ranges.

### 3.4. Management of Yield and Water Productivity

Table 3 shows a significant increase in water consumption per plant using timing system irrigation control. On the contrary, the results show a substantial increase in water use efficiency and productivity per square meter of the greenhouse using the irrigation control based on the medium moisture. Water use efficiency estimated based on the applied irrigation water is a measure of the whole system management regarding combined cultivation and irrigation system efficiency. The water use efficiency considers both the actual plant water use and the total water lost. In general, the results showed an efficiency of the automated irrigation system using the moisture sensor over the timing system, according to the cucumber fruit characteristics, plant performance, water productivity, and yield of the one-meter square and the total amount of applied irrigation water.

One of our basic targets is to maximize water productivity in the scope of the limited water supplies and energy demands by the irrigation system equipment. The only way to accomplish this is to use water effectively using modern irrigation systems [53]. The sensor-controlled irrigation system based on a precise measurement for volumetric moisture content is a useful tool to increase overall water use efficiency and improve the soilless-grown product quality [54]. The automatic irrigation system performance was evaluated by measuring the plant’s growth compared to manually irrigated plants. The growth rate shows that the higher values of total dry weights are to the plants irrigated by the automated irrigation system than the manually irrigated plant [55].

Also, Montesano et al. in [54] compared the effects of irrigation control using a timer versus a soil moisture sensor-controlled irrigation on lettuce grow in soilless media. The irrigation amounts followed the plant water needs with little or no leaching in the sensor-controlled treatments, while the lowest water use efficiency was observed, and 18% of leaching was recorded using the timing control system. Water use efficiency (WUE) is decreased with increasing the amount of irrigation water rate at one fertilization level. The amount of water of 0.0377–0.053 m${}^{3}$/plant was the best irrigation strategy for the production of drip-irrigated cultivated cucumber grown in substrate bags in spring [56]. These results coincide with the results we obtained in our research.

### 3.5. Energy Consumption

**Figure 17:**
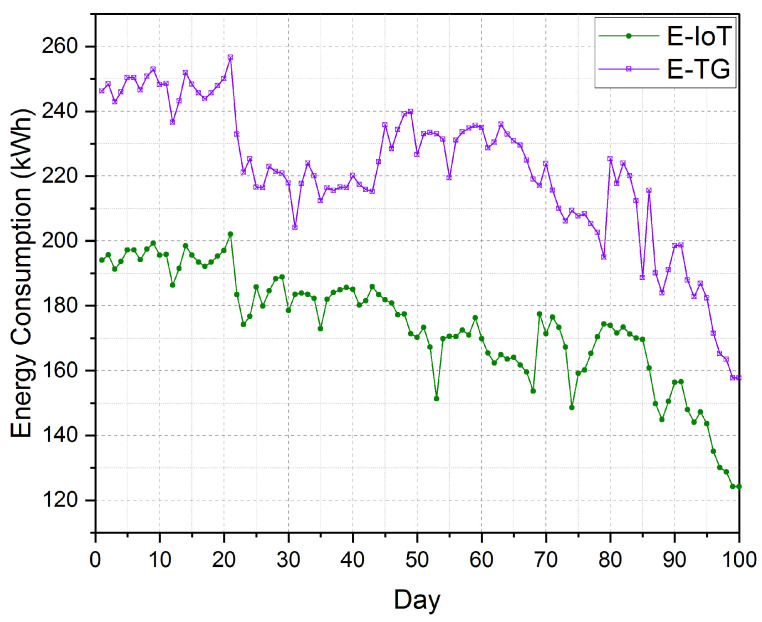
The electrical energy consumption (kWh/day) of the greenhouse with the established platform (E-IoT) against the same greenhouse under identical circumstances (E-TG).

We calculated the total electrical energy consumption for operating the greenhouse using the established IoT platform from the first week until the last week as 173,62.2 kWh. While the total electrical energy consumed by the greenhouse without the established platform was calculated in advance as 22,112 kWh, as shown in Figure 17. The average consumed electrical energy for operating the greenhouse (cooling, ventilation, and irrigation) per square meter was 7.38 kWh/m${}^{2}$. The average electrical energy productivity of cucumber was 0.412 kg/kWh under the medium moisture control system while the average electrical energy productivity was 0.342 kg/kWh.

**Figure 18:**
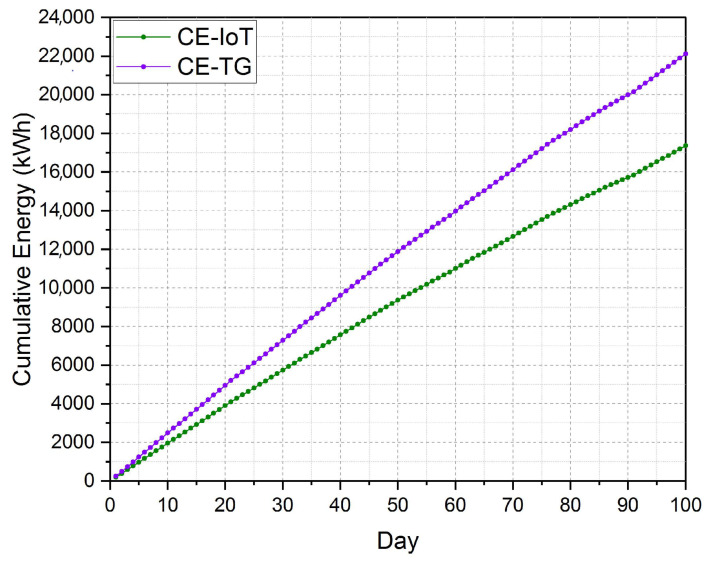
The cumulative electrical energy consumption (kWh/day) of the greenhouse with the established platform (CE-IoT) against the same greenhouse under identical circumstances (CE-TG).

In comparison with the measured total electrical energy consumed by the same greenhouse after deploying the established IoT platform, we found that we saved about 4750 kWh. Figure 18 displays the cumulative electrical energy consumption (kWh/day) of the greenhouse with the established platform against the consumption of the same greenhouse under identical circumstances. This extravagant consumption of the electrical energy of the former greenhouse is due to the inaccuracy of operating the cooling and ventilation fans. In other words, the fans and other cooling devices are turning on most of the time needless. On the other hand, the inaccuracy in timing control of turning on and off different fans leads to erratic temperatures at the recommended temperature for cucumber cultivation. Thus, the lack of a suitable climate for cucumber fruit growth leads to a delay in growth, low productivity, low water use efficiency, and high production costs. All these troubles were avoided using the established platform. Moreover, the control of irrigation water using the medium moisture sensor included in the established platform increases the productivity of the cucumber fruits per each kWh from 0.342 kg/kWh to 0.412 kg/kWh.

## 4. Conclusions

In recent years, traditional outdoor farming has been facing several challenges that require practical solutions. Smart farming, as the integration of information and communication technologies in traditional farming, could potentially address traditional farming challenges. This is made possible by incorporating IoT and digital technology in smart farming, enabling real-time monitoring, controlling, and managing the plants. As a controllable environment, greenhouses are the perfect environment to install different devices and agricultural-related equipment. This paper presented a multi-tier cloud-based IoT platform that connects sensors, actuators, and related devices placed inside the greenhouse. As a case study, we applied the established platform on cucumber fruit cultivated in a soilless medium in an integrated smart farming system. The system was established in a commercial-sized greenhouse, where we investigated the influence of the established platform on crop yield and quality. Besides, we examined the system’s impact on improving water and energy productivity through a cost-effective process, leading to increase the plants’ growth and yield. We plan to extend the current results to include two extra tiers, namely, artificial intelligence (AI) and wireless sensor networks (WSN), to enhance the quality of service in smart soilless greenhouse cultivation. The WSN-based tier will promote the current IoT tier with a long-range wide-area network module, which will be more suitable for long transmission ranges, particularly for large greenhouses. The AI-based tier will predict the evolution of different ecological parameters inside the greenhouse to prevent any common plant diseases or unwanted situations. 

## Figures and Tables

**Table 1:** Response of cucumber fruit physicochemical characteristics under different irrigation water control system of medium moisture control (MMC) and irrigation timing (ITC).

Characteristic	Method	Mean	Std. Dev.	Std. Err.	Minimum	Maximum	*p* Value
Length (cm)	MMC	14.12 A	0.69	0.17	12.50	15.60	0.00
	ITC	12.77 B	0.96	0.24	11.21	14.5	
Diameter (cm)	MMC	2.85 B	0.29	0.07	2.37	3.30	0.00
	ITC	3.17 A	0.21	0.05	2.65	3.50	
Projected Area (cm${}^{2}$)	MMC	33.89 A	5.40	1.35	25.41	43.61	0.73
	ITC	33.25 A	4.9	1.23	24.36	42.31	
Aspect ratio	MMC	0.20 B	0.02	0.00	0.17	0.23	0.00
	ITC	0.25 A	0.02	0.01	0.22	0.29	
Weight (g)	MMC	75.99 B	16.49	4.12	51.87	107.10	0.03
	ITC	86.46 A	9.07	2.27	70.00	99.80	
Volume (cm${}^{3}$)	MMC	90.46 A	19.55	4.89	57.72	121.28	0.12
	ITC	100.62 A	16.11	4.03	64.88	124.29	
Density (g/cm${}^{3}$)	MMC	0.84 A	0.08	0.02	0.64	0.95	0.37
	ITC	0.87 A	0.05	0.01	0.80	0.94	
L, Lightness	MMC	36.29 A	2.24	0.65	32.86	39.59	0.05
	ITC	38.19 A	2.24	0.65	34.76	41.49	
Hue (angle)	MMC	104.10 A	5.73	1.66	93.93	110.37	0.00
	ITC	97.73 B	3.94	1.14	91.27	102.19	
Chroma (magnitude)	MMC	18.52 B	2.91	0.84	13.84	22.50	0.00
	ITC	27.89 A	2.86	0.83	23.46	31.74	
Hardness (N)	MMC	29.27 A	5.19	1.5	20.60	37.28	0.34
	ITC	27.39 A	4.24	1.22	21.58	35.32	
Moisture content (*%*)	MMC	88.63 B	0.52	0.15	87.50	89.20	0.00
	ITC	90.63 A	0.52	0.15	89.50	91.20	
pH	MMC	5.34 A	0.11	0.03	5.12	5.54	0.90
	ITC	5.33 A	0.16	0.04	5.11	5.55	
TSS (*%*)	MMC	3.23 A	0.14	0.04	3.10	3.50	0.00
	ITC	2.93 B	0.14	0.04	2.80	3.20	

Figures with the same letter for each characteristic are non-significant at alpha values of 0.05. The data presented above indicate the average of 20 values for each characteristic.

**Table 2:** Impact of irrigation water control by medium moisture control (MMC) and irrigation timing control (ITC) on cucumber at the same condition of greenhouse microclimate control.

Parameter	Method	Mean	Std. Dev.	Std. Err.	Minimum	Maximum	*p* Value
Plant temperature (°C)	MMC	23.425 B	1.223	0.353	22.10	25.30	0.011
	ITC	24.850 A	1.276	0.368	22.20	26.70	
Chlorophyll (SPAD)	MMC	34.367 B	1.209	0.349	32.90	37.30	0.000
	ITC	31.567 A	1.328	0.383	29.50	33.80	
Number of fruit per plant	MMC	14.000 A	1.809	0.522	12.00	18.00	0.027
	ITC	12.250 B	1.815	0.524	9.00	15.00	
Yield per plant (kg/plant)	MMC	1.278 A	0.321	0.093	0.96	1.96	0.019
	ITC	1.025 B	0.039	0.039	0.80	1.20	

Figures with the same letter for each parameter are non-significant at alpha values of 0.05. The data presented above indicate the average of 10 values for each parameter.

**Table 3:** Irrigation water applied (m${}^{3}$/plant), water use efficiency (kg/m${}^{3}$), and productivity per square meter (kg/m${}^{2}$) of cucumber under different irrigation water control system of medium moisture control (MMC) and irrigation timing (ITC).

Parameter	Method	Mean	Std. Dev.	Std. Err.	Min	Max	*p* Value
Applied water per plant (m${}^{3}$/plant)	MMC	0.058 B	0.003	0.001	0.05	0.06	0.000
	ITC	0.073 A	0.010	0.003	0.06	0.10	
WUE (kg/m${}^{3}$)	MMC	19.017 A	2.456	0.709	16.30	24.45	0.000
	ITC	14.378 B	1.887	0.545	11.22	16.83	
Productivity (kg/m${}^{2}$)	MMC	4.453 A	0.804	0.232	3.64	6.51	0.004
	ITC	3.588 B	0.471	0.136	2.80	4.20	

Figures with the same letter for each parameter are non-significant at alpha values of 0.05. The data presented above indicate the average of 10 values for each parameter.

## Sample Availability

Samples of the compounds …… are available from the authors.
